# A longitudinal seroepidemiology study to evaluate antibody response to SARS-CoV-2 virus infection and vaccination in children in Calgary, Canada from July 2020 to April 2022: Alberta COVID-19 Childhood Cohort (AB3C) Study

**DOI:** 10.1371/journal.pone.0284046

**Published:** 2023-04-06

**Authors:** Emily J. Doucette, Joslyn Gray, Kevin Fonseca, Carmen Charlton, Jamil N. Kanji, Graham Tipples, Susan Kuhn, Jessica Dunn, Payton Sayers, Nicola Symonds, Guosong Wu, Stephen B. Freedman, James D. Kellner

**Affiliations:** 1 Department of Pediatrics, Cumming School of Medicine, University of Calgary, Calgary, Alberta, Canada; 2 Department of Microbiology, Immunology & Infectious Diseases, University of Calgary, Calgary, Alberta, Canada; 3 Public Health Laboratory, Alberta Precision Laboratories, Alberta, Canada; 4 Department of Pathology and Laboratory Medicine, Cumming School of Medicine, University of Calgary, Calgary, Alberta, Canada; 5 Li Ka Shing Institute of Virology, University of Alberta, Edmonton, Alberta, Canada; 6 Department of Laboratory Medicine and Pathology, Faculty of Medicine and Dentistry, University of Alberta, Edmonton, Alberta, Canada; 7 Division of Infectious Diseases, Department of Medicine, Cumming School of Medicine, University of Calgary, Calgary, Alberta, Canada; 8 Department of Medical Microbiology and Immunology, Faculty of Medicine and Dentistry, University of Alberta, Edmonton, Alberta, Canada; 9 School of Medicine, Queen’s University, Kingston, Ontario, Canada; 10 Department of Community Health Sciences, University of Calgary, Calgary, Alberta, Canada; 11 Department of Emergency Medicine, University of Calgary, Calgary, Alberta, Canada; 12 Alberta Children’s Hospital Research Institute, University of Calgary, Calgary, AB, Canada; National Center for Global Health and Medicine, JAPAN

## Abstract

**Background:**

Measurement of SARS-CoV-2 antibody seropositivity is important to accurately understand exposure to infection and/or vaccination in specific populations. This study aimed to estimate the serologic response to SARS-CoV-2 virus infection and vaccination in children in Calgary, Alberta over a two-year period.

**Methods:**

Children with or without prior SARS-CoV-2 infections, were enrolled in Calgary, Canada in 2020. Venous blood was sampled 4 times from July 2020 to April 2022 for SARS-CoV-2 nucleocapsid and spike antibodies. Demographic and clinical information was obtained including SARS-CoV-2 testing results and vaccination records.

**Results:**

1035 children were enrolled and 88.9% completed all 4 visits; median age 9 years (IQR: 5,13); 519 (50.1%) female; and 815 (78.7%) Caucasian. Before enrolment, 118 (11.4%) had confirmed or probable SARS-CoV-2. By April 2022, 39.5% of previously uninfected participants had a SARS-CoV-2 infection. Nucleocapsid antibody seropositivity declined to 16.4% of all infected children after more than 200 days post diagnosis. Spike antibodies remained elevated in 93.6% of unvaccinated infected children after more than 200 days post diagnosis. By April 2022, 408 (95.6%) children 12 years and older had received 2 or more vaccine doses, and 241 (61.6%) 5 to 11 year-old children had received 2 vaccine doses. At that time, all 685 vaccinated children had spike antibodies, compared with 94/176 (53.4%) of unvaccinated children.

**Conclusions:**

In our population, after the first peak of Omicron variant infections and introduction of COVID-19 vaccines for children, all vaccinated children, but just over one-half of unvaccinated children, had SARS-CoV-2 spike antibodies indicating infection and/or vaccination, highlighting the benefit of vaccination. It is not yet known whether a high proportion of seropositivity at the present time predicts sustained population-level protection against future SARS-CoV-2 transmission, infection or severe COVID-19 outcomes in children.

## Introduction

Following the emergence of severe acute respiratory syndrome coronavirus 2 (SARS-CoV-2), there have been over 613 million confirmed COVID-19 infections worldwide and 4.2 million in Canada [1]. Reverse-transcription polymerase chain reaction (RT-PCR) and rapid antigen assays of respiratory samples have facilitated the diagnosis of symptomatic and asymptomatic COVID-19 infections. While RT-PCR is generally accurate for identifying acute cases of COVID-19 [2], there is a role for serologic testing to identify the presence and duration of antibodies following SARS-CoV-2 infection and/or immunization.

Antibodies produced after COVID-19 infections persist for a variable time [3], with no definite level that specifies a correlate of protection [4] that will prevent reinfection. Readily available tests of IgG antibody response to SARS-CoV-2 target two of the four structural proteins of the virus; the nucleocapsid protein (N) or the receptor binding domain (RBD) of the S1 subunit of the spike protein (S) [5]. Nucleocapsid antibody is produced after a SARS-CoV-2 infection and spike antibody is produced after vaccination or infection [5]. Initial research focused on evaluating testing methods and antibody responses during acute infection and early post-infection [6–8]. Data from surveillance studies have documented that nucleocapsid antibodies persist in adults up to 41 weeks post-infection [3, 9, 10] however less data exists for children [11–18], especially data which include antibodies against virus variants. While children rarely experience moderate or severe disease [19], infections are frequent. In Canada children under 19 years comprise 21% of the population and 18% of all confirmed COVID-19 infections have occurred in this age group [20,21].

The primary objective of this study was to estimate the serial humoral response to SARS-CoV-2 virus infection and vaccination in children in Calgary, Alberta over a two-year period, including those with and without clinically apparent confirmed or probable COVID-19 infection, and those with and without COVID-19 vaccination. The secondary objective was to evaluate the duration of circulating antibodies against SARS-CoV-2 nucleocapsid and spike in children.

## Methods

### Study population

All study participants were under 18 years of age, residing in the Calgary area. Two study groups were enrolled. Electronic consent was obtained from the parents of all participating children (or directly from mature minors). The enrolment target was 1000 children. The full study methods have been previously described [22].

Group 1 included children with confirmed or probable COVID-19 infection prior to enrolment, as defined by the provincial department of health (Alberta Health) [23]. These children were identified by the provincial health services delivery agency, Alberta Health Services (AHS) and invited to participate. Group 2 included children not diagnosed with COVID-19 infections prior to enrolment, and whose families expressed interest in the study through responding to a study announcement posted on social media. Children in this group either had a prior negative PCR test for SARS-CoV-2 or were never tested.

The study received approval from the University of Calgary Conjoint Health Research Ethics Board (CHREB) (REB20-0480).

### Survey, COVID-19 diagnostic test results, and vaccination records

Participants or guardians completed an online survey following each study visit that included questions on demographic features, health history, and behaviors related to public health measures during the COVID-19 pandemic. The survey was adapted from the federal COVID-19 Immunity Task Force [24] and included questions about COVID-19 testing and vaccination. In addition to self-reporting, the results of all laboratory-conducted COVID-19 PCR tests and vaccines received were confirmed from the AHS centralized laboratory database and vaccine registry. Participants were considered immunized if one or more doses of COVID-19 immunization were received more than 14 days prior to blood sampling.

### Serology and laboratory methods

Venous blood was collected and sent to the public health laboratory. Samples were tested with two SARS-CoV-2 IgG assays. First, the Abbott ARCHITECT SARS-CoV-2 nucleocapsid IgG assay [25] was used to detect IgG antibodies against the nucleocapsid (N) protein of SARS-CoV-2. Samples with a sample calibration (S/C) value of ≥1.4 were considered positive based on manufacturer’s recommendations [25]. Second, the Abbott ARCHITECT SARS-CoV-2 spike IgG II RUO assay [26] was used to detect IgG antibodies to the receptor binding domain (RBD) of the S1 subunit of the spike protein of SARS-CoV-2. Samples with a value of ≥50.0 arbitrary units (AU)/mL were considered positive [26].

### Timing of visits

Four visits were conducted, from July 2020 and every 6 months +/- 1 month for each participant until April 2022. Visit 1 and Visit 2 occurred prior to SARS-Cov-2 variant B.1.617.2 (Delta) being declared a Variant of Concern (VOC) by the World Health Organization (WHO) in May 2021 [27], and prior to vaccine availability for those under 18. Visit 3 occurred after Delta became the dominant variant in Alberta, and after the implementation of COVID-19 vaccines for children ≥12 years, in May 2021. Visit 4 occurred following the emergence of the highly transmissible Omicron variant [27], and after vaccine implementation for children 5–11 years, in November 2021.

Children without infection at enrolment may have acquired COVID-19 infections in the intervals between visits, and both those with and without prior infection may have been reinfected. After each visit, participants were reallocated to infected (Group 3) or uninfected (Group 4) groupings depending on their updated infection status. The infected group consisted of all children who had a confirmed or probable COVID-19 infection before or during the study, and was subdivided into those with infection prior to enrolment (prior infection) and those who acquired it between Visit 1 and Visit 4 (newly infected). Children who never developed a PCR and/or rapid antigen test confirmed or probable COVID-19 infection, or never developed positive nucleocapsid antibody, throughout the study remained in the uninfected group.

By Visit 3 and Visit 4, some children from both groups had been vaccinated against COVID-19. Thus, the results were further stratified by vaccine-relevant age groups and vaccination status. The age groups included <5 years of age (ineligible for vaccine before Visit 4); 5–11 years (vaccine-eligible at Visit 4); and ≥12 years (vaccine-eligible at Visit 3).

### Data management and analysis

Electronic consent forms and surveys were hosted on a University of Calgary licensed Research Electronic Data Capture (REDCap) database [28, 29]. Serology results were compiled using Microsoft Excel 2016 and analyzed using STATA 16 [30] and GraphPad Prism 9.2.0.

The results were initially tabulated separately for participants with prior infection and those who were uninfected. For the results from Visit 3 and 4, which took place after COVID-19 vaccines were introduced for children and, for Visit 4, after the initial Omicron wave, the results from all participants were compiled and stratified by vaccination status and new infection/reinfection status. In addition, the duration of positive nucleocapsid and spike antibodies after confirmed COVID-19 infections were measured as number of days since the positive PCR test for SARS-CoV-2 antigen for all infected children for nucleocapsid antibodies, and for all unvaccinated infected children for spike antibodies.

## Results

### Participants and sampling

**Fig 1:**
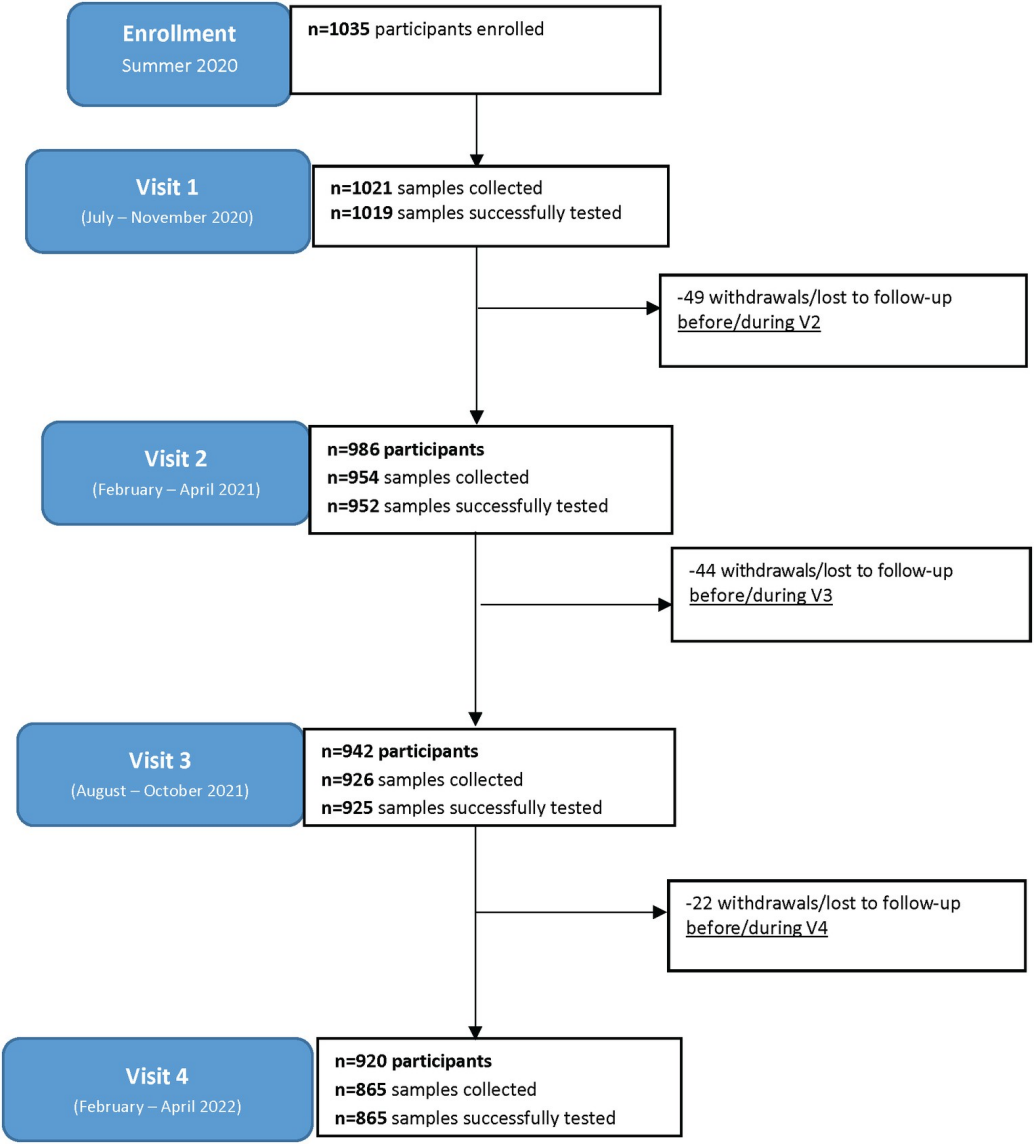
Study participation flow diagram.

A total of 1035 children were enrolled (1023 who attended Visit 1), including 118 (11.4%, 95% CI: 9.5%-13.5%) with and 917 (88.6%, 95% CI: 86.5%-90.5%) without COVID-19 infection prior to enrolment (Table 1, Fig 1). The median age of participants at enrolment was 9 years (IQR 5–13); there were 519 (50.1%, 95% CI: 47.1%-53.2%) female participants and 815 (78.7%, 95% CI: 76.1%-81.2%) self-reported Caucasian ethnicity (Table 1). Infected and uninfected participants had similar distributions of age, sex at birth, and chronic medical conditions at enrollment, however they differed significantly in ethnicity and indigenous status (Table 1). Of the participants with prior infections, 97 (82.2%, 95% CI: 74.1%-88.6%) had a positive PCR test for SARS-CoV-2, while the remaining 21 (17.8%, 95% CI: 11.3%-25.9%) were defined as probable COVID-19 infection with a history of symptoms and exposure to a confirmed case.

**Table 1:** Demographic and clinical characteristics of the sample at Visit 1.

Demographic and Clinical Features	Previously Infected at enrolment (Group 1) (n = 118)	Not infected at enrolment (Group 2) (n = 917)	Total (n = 1035)	P-value
Age at enrolment, median (IQR), years	10 (6–14)	9 (5–13)	9 (5–13)	0.1406^a^
Age group in years at enrolment, n (%), years				0.548^b^
• <5 years	23 (19.5%)	178 (19.5%)	201 (19.4%)	
• 5–11 years	48 (40.7%)	417 (45.5%	464 (44.8%)	
• 12+ years	47 (39.8%)	322 (35.1%)	369 (35.7%)	
Sex at birth, n (%)				0.329^b^
• Female	54 (45.8%)	465 (50.7%)	519 (50.1%)	
• Male	64 (54.2%)	452 (49.3%)	516 (49.9%)	
Indigenous status, n (%)				0.000^b^
• Yes	3 (2.5%)	33 (3.6%)	36 (3.5%)	
• No	105 (89.0%)	872 (95.1%)	977 (94.4%)	
• Prefer not to answer/data not collected	10 (8.5%)	12 (1.3%)	22 (2.1%)	
Ethnicity, n (%)				0.000^b^
• Caucasian	58 (49.2%)	757 (82.3%)	815 (78.7%)	
• Asian (Chinese, Filipino, Japanese, Korean, South Asian, Southeast Asian, West Asian)	24 (20.3%)	19 (2.1%)	43 (4.2%)	
• Black	4 (3.4%)	6 (0.7%)	10 (1.0%)	
• Mixed	17 (14.4%)	105 (11.5%)	122 (11.8%)	
• Other (Arab, Latin American)	2 (1.7%)	13 (1.4%)	15 (1.4%)	
• Prefer not to answer/data not collected	13 (11.0%)	17 (1.9%)	30 (2.9%)	
Chronic medical conditions, n (%)				0.344 ^b^
• Asthma	8 (6.8%)	96 (10.5%)	104 (10.0%)	
• Immune suppressed	1 (0.8%)	16 (1.7%)	17 (1.6%)	
• Chronic neurologic disorder	1 (0.8%)	11 (1.2%)	12 (1.2%)	
• Other Medical Conditions (cancer, chronic blood disorder, chronic heart disease, chronic lung disease, diabetes, hypertension, liver disease)	0 (0%)	14 (1.5%)	14 (1.4%)	
• None of the above	89 (75.4%)	595 (64.9%)	684 (66.1%)	
• Prefer not to answer/data not collected	19 (16.1%)	185 (20.2%)	204 (19.7%)	

^a^ P-value calculated using Wilcoxon signed rank test

^b^ P-values calculated using Fisher exact test

There were 3761 blood samples collected over 4 visits. The number of samples successfully tested (and percentage of total initial participants) were: 1019 at Visit 1 (98.5%, 95% CI: 97.5%-99.1%), 952 at Visit 2 (92.0%, 95% CI: 90.2%-93.6%), 925 at Visit 3 (89.4%, 95% CI: 87.3%-91.1%), and 865 at Visit 4 (83.6%, 95% CI: 81.2%-85.8%) (Fig 1). The number of participants who completed a survey at each visit (and percentage of total participants) were: 994 at Visit 1 (96.0%, 95% CI: 94.7%-97.1%), 956 at Visit 2 (92.4%, 95% CI: 90.6%-93.9%), 938 at Visit 3 (90.6%, 95% CI: 88.7%-92.3%), and 906 at Visit 4 (87.5%, 95% CI: 85.4%-89.5%). By Visit 4, 115 (11.1%, 95% CI: 9.3%-13.2%) of the total participants had withdrawn, including 49 at Visit 2, 44 at Visit 3 and 22 at Visit 4. All results prior to withdrawal or loss to follow-up were recorded.

The proportion of all vaccine eligible participants to have received at least one dose of COVID-19 vaccine was 41.8% by visit 3 and 79.2% by visit 4 (Table 2). By Visit 4 which ended in April 2022, the proportion of children immunized was 4.9% (5/102, 95% CI: 1.6%-11.1%), 79.2% (309/391, 95% CI: 74.7%-83.0%), and 96.5% (412/427, 95% CI: 94.3%-98.0%) for children aged < 5 years, 5–11 years, and ≥12 years, respectively (Table 2). Although vaccines were not yet approved for children under 5 years, 5 participants were enrolled in an mRNA vaccine clinical trial.

**Table 2:** Vaccine status of participants at Visit 3 and Visit 4 following approval for ages 11+ prior to V3 and ages 5–11 prior to V4 using age at time of visit (vaccination considered effective day 14+ post-immunization).

Visit 3 (n = 942)	Age group at Visit 3					
	<5 years (n = 121)	95% CI	5–11 years (n = 415)	95% CI	12+ years (n = 406) ^a^	95% CI
Number (%) with 0 doses	121 (100%)	(97%-100%)	394 (94.9%)	(92.3%-96.8%)	35 (8.6%)	(6.0%-11.7%)
Number (%) with 1 dose	0	(0.0%-3.0%)	0	(0.0%-0.8%)	19 (4.7%)	(2.8%-7.2%)
Number (%) with 2 doses	0	(0.0%-3.0%)	21 (5.1%)	(31.6%-76.3%)	352 (86.7%)	(83.0%-89.8%)
Number (%) with 3 doses	0	(0.0%-3.0%)	0	(0.0%-0.8%)	0	(0.0%-0.9%)
Number (%) with unknown vaccine status	0	(0.0%-3.0%)	0	(0.0%-0.8%)	0	(0.0%-0.9%)
Visit 4 (n = 920)	Age group at Visit 4					
	<5 years (n = 102)	95% CI	5–11 years (n = 391) ^c^	95% CI	12+ years (n = 427) ^c^	95% CI
Number (%) with 0 doses	97 (95.1%)	(88.9%-98.3%)	79 (20.2%)	(16.34%-24.5%)	14 (3.3%)	(1.8%-5.4%)
Number (%) with 1 dose	1 (0.98%) ^b^	(0.2%-5.3%)	68 (17.4%)	(13.7%-21.52%)	4 (0.93%)	(0.0% -2.3%)
Number (%) with 2 doses	4 (3.9%) ^b^	(1.0%-9.7%)	241 (61.6%)	(56.6%-66.4%)	354 (82.9%)	(78.9%-86.3%)
Number (%) with 3 doses	0	(0.0%-3.5%)	0	(0.0%-0.94%)	54 (12.6%)	(9.6%-16.1%)
Number (%) with unknown vaccine status	0	(0.0%-3.5%)	3 (0.8%)	(0.16%-2.2%)	1 (0.23%)	(0.01%-1.3%)

^a^ Proportion of vaccine eligible participants with ≥1 dose at Visit 3: 41.8%

^b^ 5 participants were participating in a COVID-19 vaccine clinical trial for children <5 in parallel to this study.

^c^ Proportion of vaccine eligible participants with ≥1 dose at Visit 4: 79.2%

### Incidence of COVID-19 infection during study

**Fig 2:**
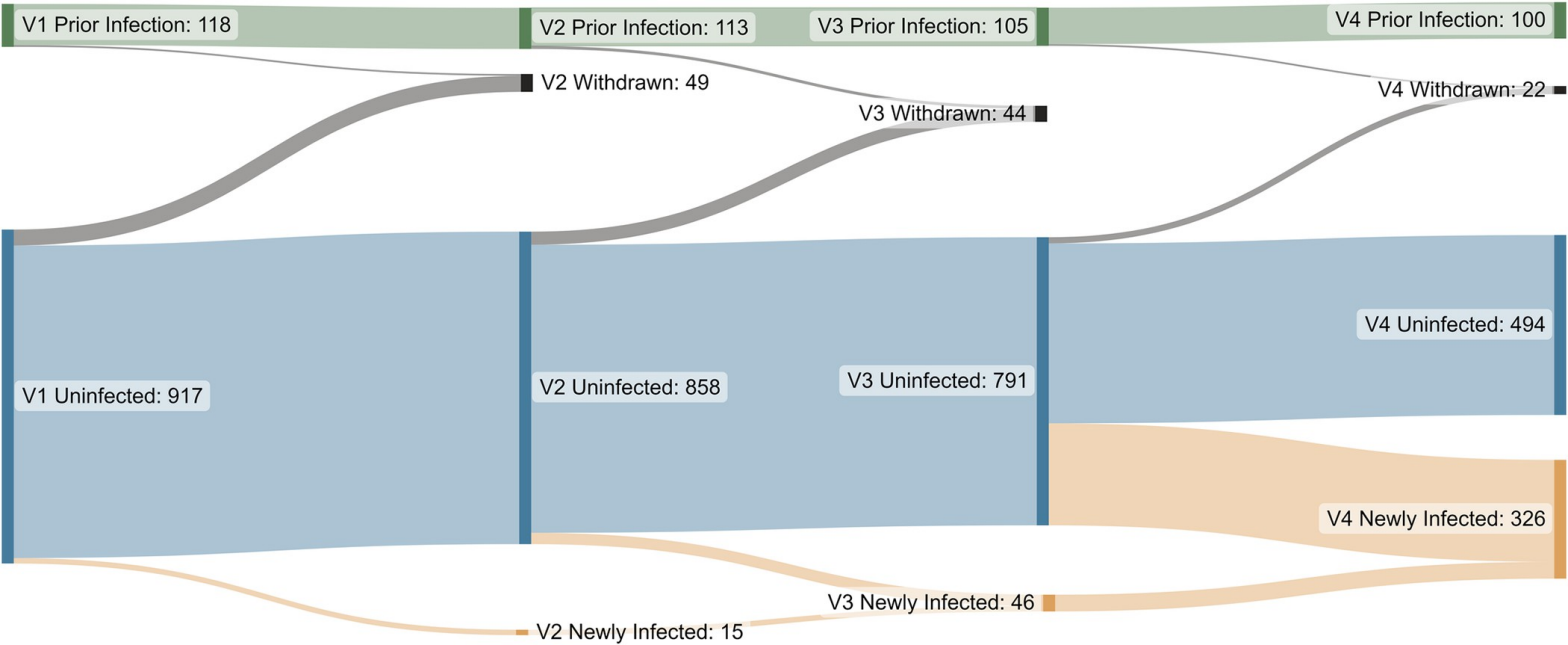
Participant reallocation between groups from Visit 1 (left-hand side) to Visit 4 (right-hand side). Participants with SARS-CoV-2 infection prior to enrolment shown in green, participants uninfected at enrolment who remained uninfected in blue, and newly infected participants who acquired SARS-CoV-2 infection between study visits in orange. There were 115 total participant withdrawals in black.

Of participants without documented prior COVID-19 infection at enrolment, the number with a newly acquired infection (positive RT-PCR, rapid test, or probable positive), was 0/917 (0%, 95% CI: 0.0%-0.4%), 15/873 (1.7%, 95% CI: 1.0%-2.8%), 31/837 (3.7%, 95% CI: 2.5%-5.2%), and 280/820 (34.1%, 30.9%-37.5%) at Visits 1, 2, 3 and 4, respectively (Fig 2). The total cumulative proportion was 39.5% by Visit 4. In addition, of 301 children who reported an infection or reinfection at Visit 4, the proportion diagnosed with only a positive rapid antigen test (185/301; 61.5%, 95% CI: 55.7%-67.0%) was higher than those with a positive PCR test with or without a rapid test (100/301; 33.2%, 95% CI: 27.9%-38.9%) or with a probable diagnosis based on exposure to a confirmed case (16/301; 5.3%, 95% CI: 3.1%-8.5%).

### COVID-19 clinical severity, reinfections, and undetected infections

Only 1 hospital admission related to a COVID-19 infection and 3 emergency department visits for symptoms associated with COVID-19 were reported (1 at Visit 1, and 2 at Visit 4). Reinfections (identified by positive PCR or rapid tests and confirmed through serology results) in children with COVID-19 infections prior to enrolment or in children who acquired COVID-19 infections after enrolment (newly infected) were reported for 2 children at Visit 3, and 21 at Visit 4 (18 with prior RT-PCR or rapid test confirmed infections and 3 with RT-PCR or rapid test confirmed infection after a previous probable infection). Neither reinfected participant at Visit 3 was immunized against SARS-CoV-2; however, 66.7% (14/21, 95% CI: 43.0%-85.4%) of participants by Visit 4 had received at least 1 dose of vaccine.

Undetected infections, defined as newly nucleocapsid antibody-positive participants without a corresponding positive COVID-19 PCR or rapid antigen test were identified in children enrolled as uninfected with 0.11% (1/909, 95% CI: 0.0%-0.6%) at Visit 1, 0.85% (7/827, 95% CI:0.3%-1.7%) at Visit 2, and 1.9% at Visit 3 (15/779, 95% CI: 1.1%-3.2%) and 16.8% (77/458, 95% CI: 13.5%-20.6%) at visit 4. The proportion of unimmunized children among participants with undetected newly positive nucleocapsid antibody was 80.0% (12/15, 95% CI: 51.9%-95.7%) at Visit 3 and 27.3% (21/77, 95% CI: 17.7%-38.6%) at Visit 4.

### Seropositivity of nucleocapsid and spike antibodies

**Fig 3:**
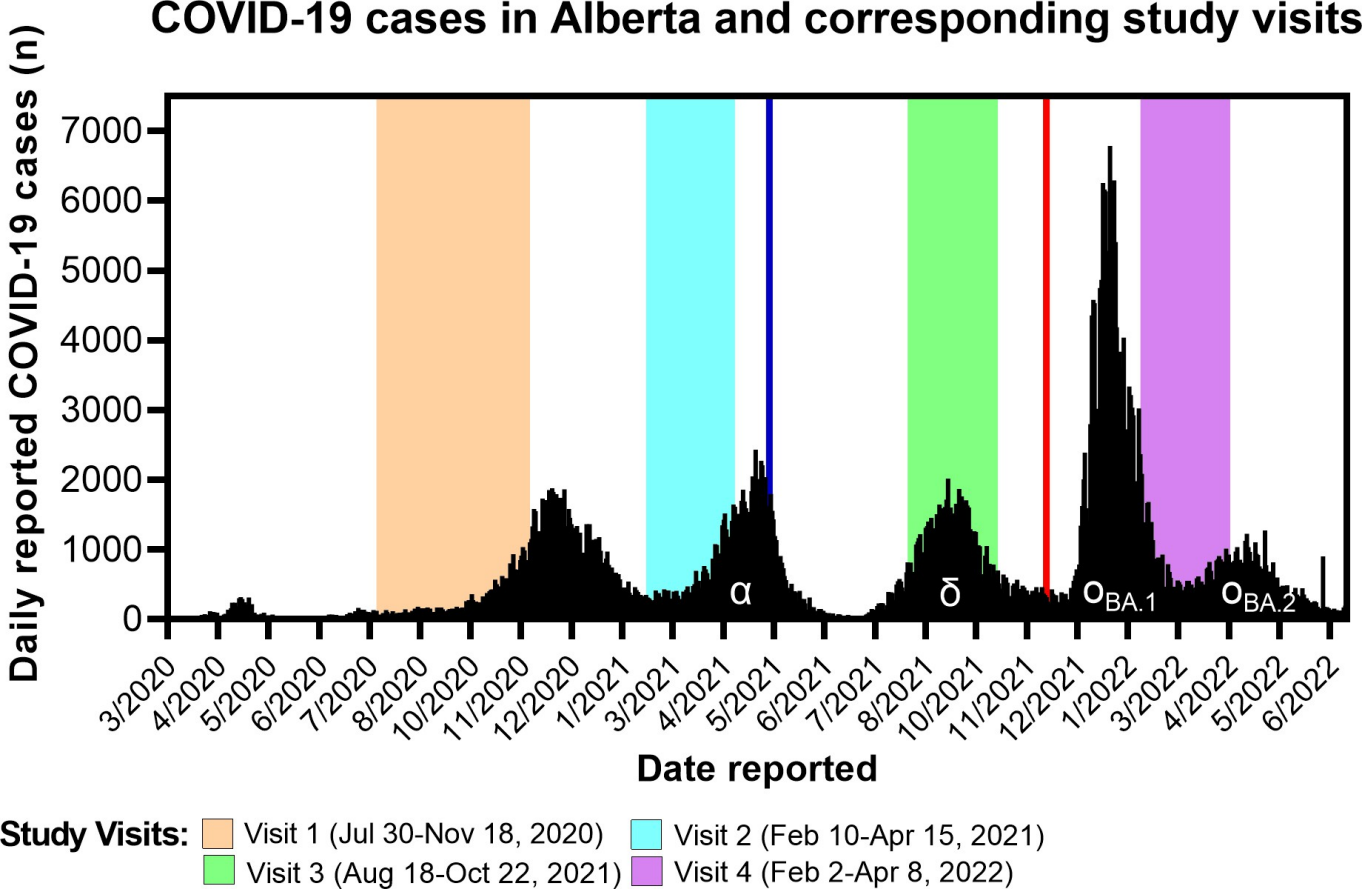
Study visit collection periods with overlay of COVID-19 pandemic curve. Date of vaccine availability for children ≥12 (May 10, 2021) indicated by vertical blue line and for children 5–11 years (November 26, 2021) indicated by vertical red line. Main variant of concern indicated by Greek letter α (Alpha B.1.1.7), δ (Delta B.1.617.2), and ο (Omicron B.1.1.529).

Table 3 shows the proportions of children with nucleocapsid and spike antibody at each visit stratified by infection status at enrolment. From Visits 1 to 3, nucleocapsid antibody seropositivity declined in those with prior infection and was low in those without infection at enrolment, then increased in both infected and uninfected groups at Visit 4, reflecting the large number of infections or reinfections with the Omicron variant (Table 3, Fig 3). Spike antibody seropositivity remained high in those with prior infection throughout the study with a rise at Visit 4 reflecting reinfection and/or vaccination. In those uninfected at enrolment, spike antibody seropositivity increased considerably at Visit 3, reflecting vaccination, and increased more at Visit 4, reflecting vaccination and new infections (Table 3).

**Table 3:** Serology results from Visits 1–4 for participants infected prior to enrolment (Group 1) and not infected at enrolment (Group 2).

Visit 1	Infected prior to enrolment (Group 1) (n = 118)		Uninfected at enrolment (Group 2) (n = 917)		Total (n = 1035)	
*SARS-Cov-2* ***Nucleocapsid*** *IgG*						
Total samples analyzed at V1	n = 110	95% CI	n = 909	95% CI	n = 1019	95% CI
• Positive	78 (70.1%)	(61.4%-79.1%)	1 (0.1%)	(0.00–0.6%)	79 (7.8%)	(6.1–9.5%)
• Negative	32 (29.9%)	(20.8%-38.5%)	908 (99.9%)	(99.3–100%)	940 (92.2%)	(90.4–93.8%)
*SARS-Cov-2* ***Spike*** *IgG*						
Total samples analyzed at V1	n = 110	95% CI	n = 902	95% CI	n = 1012	95% CI
• Positive	90 (81.8%)	(73.3%-88.5%)	6 (0.7%)	(0.2%-0.1%)	96 (9.5%)	(0.7%-11.5%)
• Negative	20 (18.2%)	(11.5%-26.7%)	896 (99.3%)	(98.6%-99.8%)	916 (90.5%)	(88.5%-92.2%)
Withdrawn Post-V1	5		44		49	
Visit 2	Group 1 (n = 113)		Group 2 (n = 873)		Total (n = 986)	
*SARS-Cov-2* ***Nucleocapsid*** *IgG*						
Total results analyzed at V2	n = 110	95% CI	n = 842	95% CI	n = 952	95% CI
• Positive	16 (14.5%)	(0.8%-22.5%)	17 (2.0%)	(0.1%-0.3%)	33 (3.5%)	(2.4%-4.8%)
• Negative	94 (85.5%)	(77.5%-91.4%)	825 (98.0%)	(96.7%-98.8%)	919 (96.5%)	(95.1%-97.6%)
*SARS-Cov-2* ***Spike*** *IgG*						
Total results analyzed at V2	n = 110	95% CI	n = 840	95% CI	n = 950	95% CI
• Positive	96 (87.3%)	(79.5%-92.9%)	27 (3.2%)	(2.1%-4.6%)	123 (12.9%)	(10.9%-15.2%)
• Negative	14 (12.7%)	(7.1%-20.4%)	813 (96.8%)	(95.4%-97.9%)	827 (87.1%)	(84.7%-89.1%)
Withdrawn Post V2	8		36		44	
Visit 3	Group 1 (n = 105)		Group 2 (n = 837)		Total (n = 942)	
*SARS-Cov-2* ***Nucleocapsid*** *IgG*						
Total results analyzed at V3	n = 101	95% CI	n = 823	95% CI	n = 924	95% CI
• Positive	5 (5.0%)	(1.6%-11.1%)	39 (4.7%)	(3.4%-6.4%)	44 (4.8%)	(3.5%-6.3%)
• Negative	96 (95.0%)	(88.8%-98.3%)	784 (95.3%)	(93.6%-96.2%)	880 (95.2%)	(93.7%-96.5%)
*SARS-Cov-2* ***Spike*** *IgG*						
Total results analyzed at V3	n = 101	95% CI	n = 822	95% CI	n = 923	95% CI
• Positive	89 (88.1%)	(80.1%-93.7%)	390 (47.4%)	(44.0%-50.9%)	479 (51.9%)	(46.6%-55.2%)
• Negative	12 (11.9%)	(6.3%-19.8%)	432 (52.6%)	(49.1%-56.0%)	444 (48.1%)	(44.8%-51.4%)
Withdrawn post V3	5		17		22	
Visit 4	Group 1 (n = 100)		Group 2 (n = 820)		Total (n = 920)	
*SARS-Cov-2* ***Nucleocapsid*** *IgG*						
Total results analyzed at V4	n = 95	95% CI	n = 770	95% CI	n = 865	95% CI
• Positive	32 (33.7%)	(24.3%-44.1%)	268 (34.8%)	(31.4%-38.3%)	300 (34.7%)	(31.5%-38.0%)
• Negative	63 (66.3%)	(55.9%-75.7%)	502 (65.2%)	(61.7%-68.6%)	565 (65.3%)	(62.0%-68.5%)
*SARS-Cov-2* ***Spike*** *IgG *						
Total results analyzed at V4	n = 95	95% CI	n = 770	95% CI	n = 865	95% CI
• Positive	93 (97.9%)	(92.6%-99.7%)	688 (89.4%)	(86.9%-91.4%)	781 (90.3%)	(88.1%-92.1%)
• Negative	2 (2.1%)	(0.02%-7.4%)	82 (10.6%)	(8.5%-13%)	84 (9.7%)	(7.8%-11.9%)

Table 4 shows the increases in nucleocapsid antibody seropositivity at Visit 4 and spike antibody seropositivity samples at Visit 3 and Visit 4 are presented for all participants, stratified by age and vaccination status. At Visit 4, the proportion of all children with positive nucleocapsid antibodies was similar in unvaccinated and vaccinated children (33.5% and 34.9%, respectively). In contrast, 100% of vaccinated children had spike antibodies by Visit 4, compared with 53.4% of unvaccinated children (Table 4).

**Table 4:** Visit 3 and Visit 4 serology results stratified by participant age and vaccine status.

**Visit 3**	**Participant Age**	**Vaccine Status at V3**	**Nucleocapsid Results, n**	**Positive, n (%)**	**95% CI**	**Negative, n (%)**	**95% CI**	**Spike Results, n**	**Positive, n (%)**	**95% CI**	**Negative, n (%)**	**95% CI**
	All participants	0 doses	537	33 (6.1%)	(4.3%-8.5%)	504 (93.9%)	(91.5%-95.7%)	537	93 (17.3%)	(14.2%-20.8%)	444 (82.7%)	(79.2%-85.8%)
		≥1 dose	387	11 (2.8%)	(1.4%-5.0%)	376 (97.2%)	(95.0%-98.6%)	386	386 (100%)	(99.0%-100%)	0	(0.0%-0.1%)
	<5 years	0 doses	120	12 (10.0%)	(5.3%-1.6%)	108 (90.0%)	(83.2%-94.7%)	119	22 (18.5%)	(12.0%-26.6%)	97 (81.5%)	(73.4%-88.0%)
		≥1 dose	N/A^a^	N/A^a^	N/A^a^	N/A^a^	N/A^a^	N/A^a^	N/A^a^	N/A^a^	N/A^a^	N/A^a^
	5–11 years	0 doses	382	15 (3.9%)	(2.2%-6.4%)	367 (96.1%)	(93.6%-97.8%)	383	56 (14.6%)	(11.2%-18.6%)	327 (85.4%)	(81.4%-88.8%)
		≥1 dose	21	0	(0.0%-1.6%)	21 (100%)	(83.9%-100%)	21	21 (100%)	(83.9%-100%)	0	(0.0%-1.6%)
	12+ years	0 doses	35	6 (17.1%)	(1.5%-5.3%)	29 (82.9%)	(6.6%-33.6%)	35	15 (42.9%)	(26.3%-60.6%)	20 (57.1%)	(39.4%-73.7%)
		≥1 dose	366	11 (3.0%)	(1.5%-5.3%)	355 (97.0%)	(94.7%-98.5%)	365	365 (100%)	(99.0%-100%)	0	(0%-1.0%)
**Visit 4**	**Participant Age**	**Vaccine status at V4**	**Nucleocapsid Results, n**	**Positive, n (%)**	**95% CI**	**Negative, n (%)**	**95% CI**	**Spike Results, n**	**Positive, n (%)**	**95% CI**	**Negative, n (%)**	**95% CI**
	All participants	0 doses	176	59 (33.5%)	(26.6%-41.0%)	117 (66.5%)	(59.0%-73.4%)	176	94 (53.4%)	(45.8%-60.9%)	82 (46.6%)	(39.1%-54.2%)
		≥1 dose	685	239 (34.9%)	(31.3%-38.6%)	446 (65.1%)	(61.4%-68.7%)	685	685 (100%)	(99.5%-100%)	0	(0%-0.5%)
	<5 years ^b^	0 doses	89	25 (28.1%)	(19.1%-38.6%)	64 (71.9%)	(61.4%-80.9%)	89	39 (43.8%)	(33.3%-54.8%)	50 (56.2%)	(0.45%,0.67%)
		≥1 dose	5	1 (20.0%)	(0.5%-71.6%)	4 (80.0%)	(28.4%-99.5%)	5	5 (100%)	(47.8%-100%)	0	(0%-52.2%)
	5–11 years	0 doses	74	26 (35.1%)	(24.4%-47.1%)	48 (64.9%)	(52.9%-75.6%)	74	46 (62.2%)	(50.1%-73.2%)	28 (37.8%)	(26.8%-49.9%)
		≥1 dose	290	98 (33.8%)	(28.4%-39.6%)	192 (66.2%)	(60.4%-71.6%)	290	290 (100%)	(98.7%-100%)	0	(0%-1.3%)
	12+ years	0 doses	13	8 (61.5%)	(31.6%-86.1%)	5 (38.5%)	(13.9%-68.4%)	13	9 (69.2%)	(38.6%-90.9%)	4 (30.8%)	(9.1%-61.4%)
		≥1 dose	390	140 (35.9%)	(31.1%-40.9%)	250 (64.1%)	(59.1%-68.9%)	390	390 (100%)	(99.1%-100%)	0	(0.0%-0.09%)

^a^ N/A indicates samples collected during time period when vaccine was not available for children aged 5–11.

^b^ 5 participants were participating in a COVID-19 vaccine clinical trial for children <5 in parallel to this study and were immunized by Visit 4.

### Nucleocapsid and Spike antibody duration

**Fig 4:**
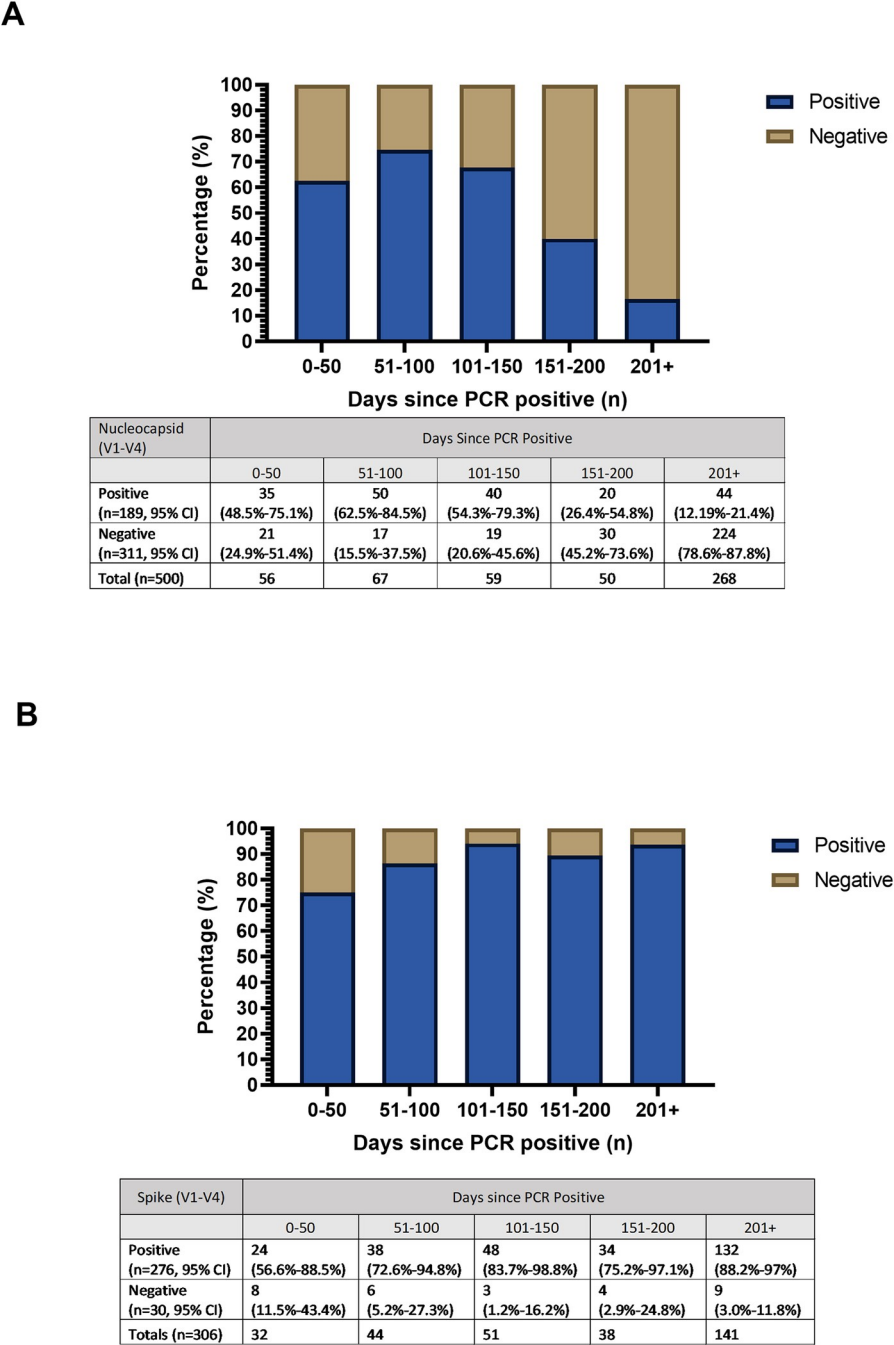
Duration of antibodies in children with confirmed SARS-CoV-2 infection. **(A)** Duration of nucleocapsid antibodies in all children from days since AHS-confirmed PCR positive to subsequent serology test(s), n = 500 samples included. (n = 19 re-infections and serology results excluded). **(B)** Duration of spike antibodies in unvaccinated children from days since AHS-confirmed PCR positive to subsequent serology test(s), n = 306 samples included. (n = 19 samples excluded from re-infections, n = 61 and n = 131 samples excluded at V3 and V4 respectively following vaccination of participants).

Most, but not all children with RT-PCR confirmed SARS-CoV-2 infections had positive nucleocapsid and spike antibodies at some point in time. More than 150 days after diagnosis, the proportion with nucleocapsid antibodies among all children with PCR-confirmed infection declined (Fig 4A). In contrast, the proportion with spike antibodies among unvaccinated infected children did not decline after more than 200 days (Fig 4B) and all 24 children measured from 451 days to more than 700 days after infection remained anti-spike antibody positive.

## Discussion

We completed SARS-CoV-2 IgG serology testing on over 1000 children on four occasions from July 2020—April 2022, sampling through successive waves of the pandemic. Before introduction of COVID-19 vaccines for children and youth under 18 years of age, and before the Omicron wave starting in late December 2021, few children seroconverted against SARS-CoV-2 from symptomatic or asymptomatic infection. Seropositivity data on pediatric populations is limited, however our results indicating low levels of undetected infection are similar to Canadian adult literature results from the same period before the Omicron waves [31].

By April 2022, after the start of COVID-19 vaccine programs for children ≥5 years and the first peak of Omicron infections, more than one-third of our sample had serologic evidence of prior infection and 90% had serologic evidence of prior infection and/or immunization. Multiple studies have shown comparably high rates of symptomatic infection following Omicron spread [32, 33] due to its increased transmissibility and the decreased response from vaccines, although risk for hospitalization may be decreased and milder outcomes are reported [34].

Since infections occurred in about equal proportions in both unvaccinated and vaccinated children during the period of Omicron variant predominance, our estimate of COVID-19 infection, on the basis of nucleocapsid antibody positivity, may be generalizable to the whole population of our province. This is because symptomatic, but not severe, COVID-19 infection is not well prevented after the primary vaccine series, especially after more than 6 months [35]. Thus, the estimate of overall seropositivity based on spike antibody positivity, is higher than for the whole population. This is because a higher proportion of children in the study were vaccinated compared to the general population, and overall seropositivity is considerably higher in vaccinated children [36]. Thus, our findings highlight the importance of vaccination, in addition to acquired infection, to achieve high levels of seropositivity in children.

This study was able to distinguish children who were seropositive on the basis of SARS-CoV-2 vaccination only, infection only or both vaccination and infection, through measurement of both nucleocapsid antibodies acquired post-SARS-CoV-2 infection and spike antibodies from COVID-19 infection and/or immunization [37]. While cumulative nucleocapsid IgG positivity (indicating SARS-CoV-2 infection) remained low at Visit 3 in the autumn of 2021, spike antibody positivity was much higher by then, reflecting rapid uptake of vaccine in the eligible target population at that time [36].

SARS-CoV-2 nucleocapsid IgG antibodies were not found in a considerable minority of children in this study even soon after their infection (within 100 days) and the proportion with nucleocapsid antibodies declined to a low level after 150 days, in contrast to spike IgG antibodies, which stayed positive in nearly all children with and without vaccination or reinfection. This is consistent with findings from adult studies describing a decline in antibody response to the nucleocapsid protein over time [5, 38]. While a small proportion of participants in this study remained nucleocapsid antibody positive more than 750 days post infection, nearly all seroconverted to negative within 200 days post-infection. In contrast, nearly all SARS-CoV-2 infected unvaccinated participants in our cohort still had spike antibodies throughout the study period, similar to residual blood studies that estimated anti-spike antibodies persistence in more than 95% of participants up to 200 days post infection [10].

Strengths of our study include the longitudinal study design with high participant retention and multiple measurements over multiple waves of the COVID-19 pandemic and the introduction of vaccines, all stratified by age and COVID-19 infection and/or vaccination status. There are multiple reports of point estimates of seropositivity against SARS-CoV-2 in different populations [39, 40]. Taken together, these show a consistent picture of a gradual rise in population seropositivity during 2020, followed by a sharp rise in 2021 as vaccines were introduced and then levelling off at seropositivity rates nearing 100% in late 2021 into early 2022 after the first Omicron variant wave. However, there are very few published longitudinal studies of repeated SARS-CoV-2 serological measure in children over time, which also include data on incidence of infections and vaccine uptake. Most such studies were conducted before vaccines were introduced [12–18], followed children for less than one year [12–15, 18] and were conducted before the Omicron wave of the pandemic [12–18]. While serial point prevalence surveys using residual blood sampling have been able to document the rise in seropositivity over the course of the pandemic and were used to inform public health interventions, the clinical and demographic data from these samples was limited and cannot readily distinguish between seropositivity from infection, vaccination, or both [3, 12, 41, 42].

Limitations of this study include the convenience sampling method of recruitment, as those who are willing to participate in research may be more likely to follow preventative health measures [43], and therefore may have been less likely to be infected with SARS-CoV-2. The sample size was smaller than some comparable serology studies [13, 41], most children enrolled in the study were healthy with few underlying conditions, and the initially uninfected study group was not as ethnically diverse as the group who had COVID-19 infections prior to enrolment.

Our results highlight the importance of seropositivity studies as essential tools for monitoring the COVID-19 pandemic [38, 44]. The pandemic has evolved significantly including the emergence of multiple variant strains of SARS-CoV-2 and the introduction of COVID-19 vaccines. The reduction in diagnostic PCR testing for active infection in our region and elsewhere may have contributed to an underestimation of the number of SARS-CoV-2 infections, highlighting the need for serologic testing to accurately measure proportions infected.

Although it is not yet clear what a high level of seropositivity in a population means in terms of individual protection against future infections and/or severe disease outcomes, some policy makers and health officials now consider that children are largely protected against COVID-19, at least for the present. This is arguable, given that based on our data, a considerable proportion of children in some settings have not had COVID-19 infections, and that uptake of vaccine in young children is lower than for older pediatric groups, and emerging variants continue to contribute towards both first infections and reinfections.

Similarly, efforts to establish an immunological correlate of protections against the outcomes of SARS-CoV-2 infections have progressed with evidence to support use of the proportion of persons in a population with a threshold level of binding antibody against spike protein, standardized to make different assays comparable, as indicative of level of protection against outcomes of relevance e.g., protection against severe disease [45]. Threshold binding antibody levels are highly correlated with neutralizing antibody assays, which have also been commonly used to estimate correlates of protection [45, 46]. Therefore, future SARS-CoV-2 serology studies can provide essential information on the incidence and prevalence of SARS-CoV-2 seropositivity, the durability of antibody responses after infection and/or vaccination, and estimate population level correlates of protection against relevant outcomes.

## References

[1] World Health Organization. WHO Coronavirus (COVID-19) Dashboard [28SEP2022]. Available from: https://covid19.who.int/.

[2] Corman VM, Landt O, Kaiser M, Molenkamp R, Meijer A, Chu DK, et al. Detection of 2019 novel coronavirus (2019-nCoV) by real-time RT-PCR. Euro Surveill. 2020;25(3). 10.2807/1560-7917.ES.2020.25.3.2000045 PMC6988269 31992387

[3] Charlton CL, Nguyen LT, Bailey A, Fenton J, Plitt SS, Marohn C, et al. Pre-Vaccine Positivity of SARS-CoV-2 Antibodies in Alberta, Canada during the First Two Waves of the COVID-19 Pandemic. Microbiol Spectr. 2021;9(1):e0029121. doi: 10.1128/Spectrum.00291-21 34406813 PMC8552659

[4] Perry J, Osman S, Wright J, Richard-Greenblatt M, Buchan SA, Sadarangani M, et al. Does a humoral correlate of protection exist for SARS-CoV-2? A systematic review. PLoS One. 2022;17(4):e0266852. doi: 10.1371/journal.pone.0266852 35395052 PMC8993021

[5] Fenwick C, Croxatto A, Coste AT, Pojer F, Andre C, Pellaton C, et al. Changes in SARS-CoV-2 Spike versus Nucleoprotein Antibody Responses Impact the Estimates of Infections in Population-Based Seroprevalence Studies. J Virol. 2021;95(3). doi: 10.1128/JVI.01828-20 33144321 PMC7925109

[6] Chew KL, Tan SS, Saw S, Pajarillaga A, Zaine S, Khoo C, et al. Clinical evaluation of serological IgG antibody response on the Abbott Architect for established SARS-CoV-2 infection. Clin Microbiol Infect. 2020;26(9):1256 e9– e11. doi: 10.1016/j.cmi.2020.05.036 32531475 PMC7282795

[7] Bryan A, Pepper G, Wener MH, Fink SL, Morishima C, Chaudhary A, et al. Performance Characteristics of the Abbott Architect SARS-CoV-2 IgG Assay and Seroprevalence in Boise, Idaho. J Clin Microbiol. 2020;58(8). doi: 10.1128/JCM.00941-20 32381641 PMC7383515

[8] Paiva KJ, Grisson RD, Chan PA, Huard RC, Caliendo AM, Lonks JR, et al. Validation and performance comparison of three SARS-CoV-2 antibody assays. J Med Virol. 2021;93(2):916–23. doi: 10.1002/jmv.26341 32710669

[9] Feng C, Shi J, Fan Q, Wang Y, Huang H, Chen F, et al. Protective humoral and cellular immune responses to SARS-CoV-2 persist up to 1 year after recovery. Nature Communications. 2021;12(1):4984. doi: 10.1038/s41467-021-25312-0 34404803 PMC8370972

[10] Grandjean L, Saso A, Torres Ortiz A, Lam T, Hatcher J, Thistlethwayte R, et al. Long-Term Persistence of Spike Protein Antibody and Predictive Modeling of Antibody Dynamics After Infection With Severe Acute Respiratory Syndrome Coronavirus 2. Clin Infect Dis. 2022;74(7):1220–9. doi: 10.1093/cid/ciab607 34218284 PMC8994590

[11] Yung CF, Saffari SE, Mah SYY, Tan NWH, Chia WN, Thoon KC, et al. Analysis of Neutralizing Antibody Levels in Children and Adolescents Up to 16 Months After SARS-CoV-2 Infection. Jama Pediatr. 2022. doi: 10.1001/jamapediatrics.2022.3072 36036929 PMC9425283

[12] Waterfield T, Watson C, Moore R, Ferris K, Tonry C, Watt A, et al. Seroprevalence of SARS-CoV-2 antibodies in children: a prospective multicentre cohort study. Arch Dis Child. 2021;106(7):680–6. doi: 10.1136/archdischild-2020-320558 33172887

[13] Ulyte A, Radtke T, Abela IA, Haile SR, Ammann P, Berger C, et al. Evolution of SARS-CoV-2 seroprevalence and clusters in school children from June 2020 to April 2021: prospective cohort study Ciao Corona. Swiss Med Wkly. 2021;151:w30092. doi: 10.4414/smw.2021.w30092 34797618

[14] Kleynhans J, Tempia S, Wolter N, von Gottberg A, Bhiman JN, Buys A, et al. SARS-CoV-2 Seroprevalence in a Rural and Urban Household Cohort during First and Second Waves of Infections, South Africa, July 2020-March 2021. Emerg Infect Dis. 2021;27(12):3020–9. doi: 10.3201/eid2712.211465 34477548 PMC8632160

[15] Kirsten C, Unrath M, Lück C, Dalpke AH, Berner R, Armann J. SARS-CoV-2 seroprevalence in students and teachers: a longitudinal study from May to October 2020 in German secondary schools. BMJ Open. 2021;11(6):e049876. doi: 10.1136/bmjopen-2021-049876 34112645 PMC8193693

[16] Tagarro A, Sanz-Santaeufemia FJ, Grasa C, Cobos E, Yebra J, Alonso-Cadenas JA, et al. Dynamics of Reverse Transcription-Polymerase Chain Reaction and Serologic Test Results in Children with SARS-CoV-2 Infection. J Pediatr. 2022;241:126–32.e3. doi: 10.1016/j.jpeds.2021.09.029 34571020 PMC8463102

[17] Beretta O, Casati Pagani S, Lazzaro M, Merlani G, Bouvier Gallacchi M. Seroprevalence of the SARS-CoV-2 virus in the population of the southern Switzerland (Canton Ticino)—cohort study, results at 12 months. Swiss Med Wkly. 2021;151:w30116. doi: 10.4414/smw.2021.w30116 34964622

[18] Zinszer K, McKinnon B, Bourque N, Pierce L, Saucier A, Otis A, et al. Seroprevalence of SARS-CoV-2 Antibodies Among Children in School and Day Care in Montreal, Canada. JAMA Netw Open. 2021;4(11):e2135975. doi: 10.1001/jamanetworkopen.2021.35975 34812845 PMC8611475

[19] King JA, Whitten TA, Bakal JA, McAlister FA. Symptoms associated with a positive result for a swab for SARS-CoV-2 infection among children in Alberta. CMAJ. 2021;193(1):E1–E9. doi: 10.1503/cmaj.202065 33234533 PMC7774482

[20] Government of Canada. COVID-19 daily epidemiology update. [28SEP22]. Available from: https://health-infobase.canada.ca/covid-19/epidemiological-summary-covid-19-cases.html?stat=num&measure=total&map=hr#a2.

[21] Statistics Canada. Population estimates on July 1st, by age and sex. [updated 28SEP2022]. Available from: https://www150.statcan.gc.ca/t1/tbl1/en/tv.action?pid=1710000501.

[22] Alberta Childhood COVID-19 Cohort (AB3C) Aim 3: Longitudinal Sero-Epidemiology Study First Interim Report January 31, 2021. 2021 [cited 29AUG2022]. Available from: https://prism.ucalgary.ca/handle/1880/113084.

[23] Government of Alberta. Alberta Public Health Disease Management Guidelines (November 2021). [03DEC2021]. Available from: https://open.alberta.ca/publications/coronavirus-covid-19.

[24] COVID-19 Immunity Task Force. Tools & Information for Researchers. 2021. Available from: https://www.covid19immunitytaskforce.ca/task-force-research-2/.

[25] Abbott. SARS-CoV-2 IgG ARCHITECT—Instructions for Use 2022 [30SEP2022]. Available from: https://www.fda.gov/media/137383/download.

[26] Abbott. SARS-CoV-2 IgG II ARCHITECT—Instructions for Use 2022 [30SEP2022]. Available from: https://www.fda.gov/media/146371/download.

[27] World Health Organization. Tracking SARS-CoV-2 Variants [03DEC2021]. Available from: https://www.who.int/en/activities/tracking-SARS-CoV-2-variants/. 37184162

[28] Harris PA, Taylor R, Thielke R, Payne J, Gonzalez N, Conde JG. Research electronic data capture (REDCap)—a metadata-driven methodology and workflow process for providing translational research informatics support. J Biomed Inform. 2009;42(2):377–81. doi: 10.1016/j.jbi.2008.08.010 18929686 PMC2700030

[29] Harris PA, Taylor R, Minor BL, Elliott V, Fernandez M, O’Neal L, et al. The REDCap consortium: Building an international community of software platform partners. J Biomed Inform. 2019;95:103208. doi: 10.1016/j.jbi.2019.103208 31078660 PMC7254481

[30] STATA Software (StataCorp). Stata Statistical Software: Release 16. College Station, TX: StataCorp LLC.2019

[31] Kanji JN, Proctor DT, Stokes W, Berenger BM, Silvius J, Tipples G, et al. Multicenter Postimplementation Assessment of the Positive Predictive Value of SARS-CoV-2 Antigen-Based Point-of-Care Tests Used for Screening of Asymptomatic Continuing Care Staff. J Clin Microbiol. 2021;59(11):e0141121. doi: 10.1128/JCM.01411-21 34288728 PMC8525574

[32] Poon RW, Chan BP, Chan WM, Fong CH, Zhang X, Lu L, et al. SARS-CoV-2 IgG seropositivity after the severe Omicron wave of COVID-19 in Hong Kong. Emerg Microbes Infect. 2022;11(1):2116–9. doi: 10.1080/22221751.2022.2106899 35880656 PMC9448364

[33] Mari A, Garancini N, Barcellini L, Zuccotti GV, Alberti L, Gaia P, et al. SARS-CoV-2 Seroprevalence Among School-Age Children in Milan: How Has It Changed With the Fourth Pandemic Wave? Pediatr Infect Dis J. 2022;41(8):e344–e5. doi: 10.1097/INF.0000000000003583 35622423 PMC9281415

[34] Wang L, Berger NA, Kaelber DC, Davis PB, Volkow ND, Xu R. Comparison of outcomes from COVID infection in pediatric and adult patients before and after the emergence of Omicron. medRxiv. 2022. doi: 10.1101/2021.12.30.21268495 35018384 PMC8750707

[35] Higdon MM, Baidya A, Walter KK, Patel MK, Issa H, Espie E, et al. Duration of effectiveness of vaccination against COVID-19 caused by the omicron variant. Lancet Infect Dis. 2022;22(8):1114–6. doi: 10.1016/S1473-3099(22)00409-1 35752196 PMC9221361

[36] Government of Alberta. COVID-19 Alberta statistics [28SEP2022]. Available from: https://www.alberta.ca/stats/covid-19-alberta-statistics.htm.

[37] Kanji JN, Bailey A, Fenton J, Ling SH, Rivera R, Plitt S, et al. Detection of SARS-CoV-2 antibodies formed in response to the BNT162b2 and mRNA-1237 mRNA vaccine by commercial antibody tests. Vaccine. 2021;39(39):5563–70. doi: 10.1016/j.vaccine.2021.08.022 34454782 PMC8354789

[38] He Z, Ren L, Yang J, Guo L, Feng L, Ma C, et al. Seroprevalence and humoral immune durability of anti-SARS-CoV-2 antibodies in Wuhan, China: a longitudinal, population-level, cross-sectional study. Lancet. 2021;397(10279):1075–84. doi: 10.1016/S0140-6736(21)00238-5 33743869 PMC7972311

[39] COVID-19 Immunity Task Force. Serotracker. [29SEP2022]. Available from: https://www.covid19immunitytaskforce.ca/serotracker/.

[40] Centers for Disease Control and Prevention. COVID Data Tracker Atlanta, GA: US Department of Health and Human Services. 2022. Available from: https://covid.cdc.gov/covid-data-tracker.

[41] Indenbaum V, Lustig Y, Mendelson E, Hershkovitz Y, Glatman-Freedman A, Keinan-Boker L, et al. Under-diagnosis of SARS-CoV-2 infections among children aged 0–15 years, a nationwide seroprevalence study, Israel, January 2020 to March 2021. Euro Surveill. 2021;26(48). 10.2807/1560-7917.ES.2021.26.48.2101040 PMC8641070 34857069

[42] Couture A, Lyons BC, Mehrotra ML, Sosa L, Ezike N, Ahmed FS, et al. Severe Acute Respiratory Syndrome Coronavirus 2 Seroprevalence and Reported Coronavirus Disease 2019 Cases in US Children, August 2020-May 2021. Open Forum Infect Dis. 2022;9(3):ofac044. doi: 10.1093/ofid/ofac044 35198651 PMC8860150

[43] Ganguli M, Lytle ME, Reynolds MD, Dodge HH. Random versus volunteer selection for a community-based study. J Gerontol A Biol Sci Med Sci. 1998;53(1):M39–46. doi: 10.1093/gerona/53a.1.m39 9467432

[44] Gudbjartsson DF, Norddahl GL, Melsted P, Gunnarsdottir K, Holm H, Eythorsson E, et al. Humoral Immune Response to SARS-CoV-2 in Iceland. N Engl J Med. 2020;383(18):1724–34. doi: 10.1056/NEJMoa2026116 32871063 PMC7494247

[45] Goldblatt D, Fiore-Gartland A, Johnson M, Hunt A, Bengt C, Zavadska D, et al. Towards a population-based threshold of protection for COVID-19 vaccines. Vaccine. 2022;40(2):306–15. doi: 10.1016/j.vaccine.2021.12.006 34933765 PMC8673730

[46] Gilbert PB, Donis RO, Koup RA, Fong Y, Plotkin SA, Follmann D. A Covid-19 Milestone Attained—A Correlate of Protection for Vaccines. N Engl J Med. 2022;387(24):2203–6. doi: 10.1056/NEJMp2211314 36507702

